# Enhanced response to dabrafenib plus trametinib in a patient with *BRAF* ^V600E^ lung cancer harboring an *RNF43* variant of unknown significance: a case report

**DOI:** 10.3389/fonc.2025.1600457

**Published:** 2025-07-15

**Authors:** Ettore D’Argento, Antonio Vitale, Jacopo Russo, Angelo Minucci, Alessandra Cancellieri, Alessio Stefani, Federico Monaca, Guido Horn, Denis Occhipinti, Paola Troisi, Alessandro Scala, Sara Polidori, Francesco D’Argento, Mariantonietta Di Salvatore, Emilio Bria, Giampaolo Tortora

**Affiliations:** ^1^ Comprehensive Cancer Center, Medical Oncology Department, Fondazione Policlinico, Universitario Agostino Gemelli IRCCS, Rome, Italy; ^2^ Faculty of Medicine and Surgery, Università Cattolica del Sacro Cuore, Rome, Italy; ^3^ Departmental Unit of Molecular and Genomic Diagnostics, Fondazione Policlinico Universitario Agostino Gemelli IRCCS, Rome, Italy; ^4^ Pathology Unit, Fondazione Policlinico Universitario Agostino Gemelli IRCCS, Rome, Italy; ^5^ UOC Radiologia e Neuroradiologia, Dipartimento di Diagnostica per Immagini, Radioterapia Oncologica ed Ematologia, Fondazione Policlinico Universitario A. Gemelli IRCCS, Roma, Italy; ^6^ Medical Oncology, Ospedale Isola Tiberina – Gemelli Isola, Rome, Italy

**Keywords:** non-small-cell lung cancer, dabrafenib, trametinib, BRAF V600E, RNF4, case report

## Abstract

**Background:**

Literature evidence reports that *RNF43* (ring finger protein 43) gene mutations could serve as predictive biomarkers of response to certain anti-cancer therapies. To delve deeper into the specific role of *RNF43* mutations in lung cancer and their relevance to therapy response, we provide the first report of marked efficacy of the dabrafenib and trametinib therapeutic combination in a patient with microsatellite-stable (MSS) non-small-cell lung cancer (NSCLC) with *BRAF^V600^* and *RNF43* mutations.

**Case description:**

An 85-year-old patient was diagnosed with NSCLC with the presence of MSS, *BRAF* ^V600E^ and *RNF43* mutations. The patient started the combination treatment with dabrafenib and trametinib, soon reporting an overall clinical benefit. A contrast-enhanced cranio–thorax–abdomen CT scan performed after 1 month of therapy reported a sharp reduction in lung cancer and hilo-mediastinal lymphadenomegaly; the central colliquation of the left adrenal metastasis was also reported. After 9 months of therapy, the cranio-thorax-abdomen CT scan with contrast medium confirmed the reduction of the adenocarcinoma, with residual scarring component; the right adrenal lesion was not visible, and the contralateral lesion was stable. At the last follow-up (February 2024), the global clinical condition of the patient was good; she was autonomous, and oxygen therapy was not necessary.

**Conclusions:**

Our clinical case represents the first report of marked efficacy of the dabrafenib–trametinib combination reported in an 85-year-old patient diagnosed with NSCLC with the presence of MSS, *BRAF* ^V600E^ and *RNF43* mutations. This supports the hypothesis on the relevance of *RNF43* mutations in predicting the clinical benefit of targeted therapies and in modulating the anti-tumor activity of anti-BRAF therapies, suggesting that *RNF43* mutations represent a promising biomarker that warrants further validation for its potential to help prioritize therapy combinations in selected lung cancer patients.

## Introduction

One of the most important advances of modern oncology is the shift from an organ-centric concept guiding treatment choice towards deep molecular analysis, driving a personalized approach. In particular, the identification of molecular tumor dependencies that can be targeted with available treatments is on the basis of precision medicine in cancer patients (1).

*BRAF* ^V600E^ mutation occurs in 1–2% of lung adenocarcinomas and acts as an oncogenic driver, activating the MAPK/ERK signaling pathway and promoting cancer cell growth and survival (2, 3). In patients with *BRAF* ^V600E^-mutant non-small-cell lung cancer (NSCLC), dabrafenib (BRAF inhibitor) and trametinib (MEK inhibitor) combination therapy has shown substantial anti-tumor activity and improved treatment outcomes compared with previous observations in patients who primarily received standard-of-care chemotherapy, thanks to its activity on the different components of the MAPK/ERK pathway (4–6). While dabrafenib and trametinib combination therapy represents a valuable treatment option for NSCLC patients with *BRAF* ^V600E^ mutations, further research is needed to identify additional predictive factors and optimize patient selection in this setting.

RNF43 (ring finger protein 43), an E3 ligase with a RING finger motif for protein–protein interaction, can act as a negative regulator of the WNT signaling pathway by promoting the ubiquitination and degradation of WNT receptors (such as Frizzled) on the cell surface. This action reduces the WNT signaling activity, helping to maintain cellular homeostasis and promoting the tumor suppression function of RNF43 (7). The *RNF43* gene is often mutated in various types of cancers, including lung cancer (8). These mutations can lead to increased WNT signaling and the consequent dysregulation of important cellular processes, such as proliferation, apoptosis, and cell cycle control, thereby contributing to cancer development and progression (9).

Literature evidence reports that *RNF43* mutations could serve as predictive biomarkers of response to certain anti-cancer therapies. For example, there is evidence that some *RNF43* mutations may influence the response to targeted therapies, such as anti-EGFR (epidermal growth factor receptor) or anti-BRAF drugs, in specific subsets of patients (10). In particular, Elez and collaborators demonstrated that inactivating *RNF43* mutations are predictive of response to anti-BRAF drugs in microsatellite-stable (MSS) colorectal cancer patients (10).

Dabrafenib plus trametinib is currently approved and represents the standard of care for first-line treatment in NSCLC patients with *BRAF^V600E^* mutations. To delve deeper into the specific role of *RNF43* mutations in lung cancer and their relevance to therapy response, we provide the first report of marked efficacy of the dabrafenib and trametinib therapeutic combination in a patient with MSS NSCLC with *BRAF^V600^* and *RNF43* mutations. The study was conducted within the protocol approved by the Ethics Committee of “Agostino Gemelli” University Hospital Foundation (Prot. N. 0001151/24 –28/06/2024). The patient provided written informed consent. We present the following case in accordance with the CARE reporting checklist.

## Case presentation

An 85-year-old female patient presented in December 2022 with a worsening dry cough and dyspnea, with severe limitation of daily activities; the Eastern Cooperative Oncology Group (ECOG) performance status (PS) was 2. The patient began oxygen therapy at 6 l/minute during daylight hours, at night rest, and during physical activity. The patient was a former smoker (moderate cigarette consumption, five packs/year) and denied occupational exposures among the risk factors. Some comorbidities were reported, namely arterial hypertension, anxiety-depressive syndrome, and spondylolisthesis.

**Figure 1:**
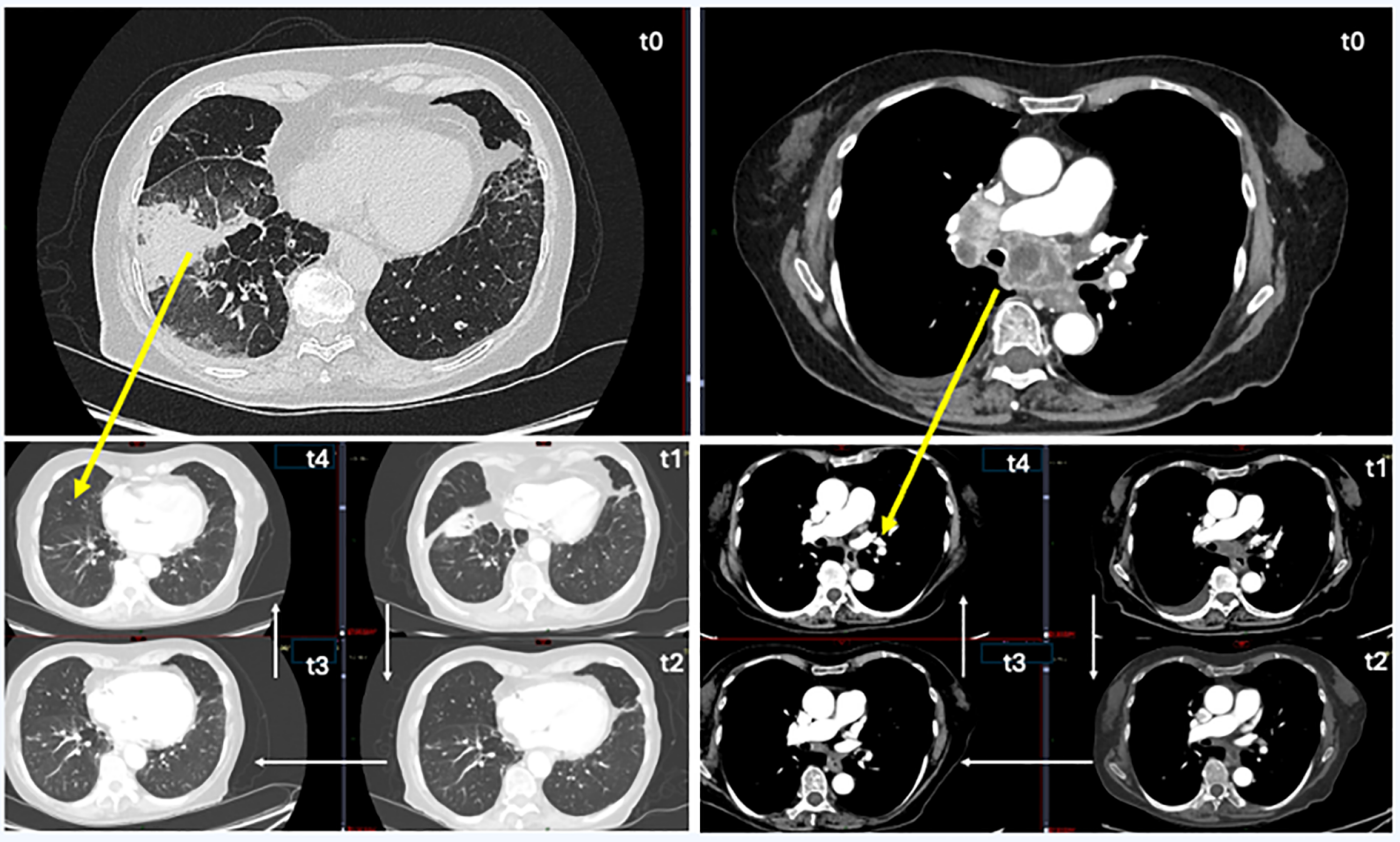
CT chest follow-up images. On the left, images with lung-window CT view, and on the right, images with mediastinal window. T0 (time 0): before therapy, pulmonary neoplasia in the right lung (lower lung lobe) and mediastinal subcarinal lymphatic localizations. T1 (after 3 months): start the reduction of lung parenchymal neoplastic mass and subcarinal lymphatic localizations. T2 (after 5 months): unrecognizable lung parenchymal neoplasm, further reduction of lymphatic localizations and their contact with the airways and pulmonary arteries. T3 (after 9 months): unrecognizable lung parenchymal neoplasm, marked reduction of subcarinal lymphatic localizations. T4 (after 12 months): no radiological evidence of oncological disease, optimal response to treatment.

The patient underwent a comprehensive staging workup that included contrast-enhanced cranio-thorax-abdomen computed tomography (CT), which documented the primary lung lesion (neoformation in the right lower lobe of the lung), hilo-mediastinal lymphadenopathy, and a left adrenal metastasis (**Figure 1**, T0).

**Figure 2:**
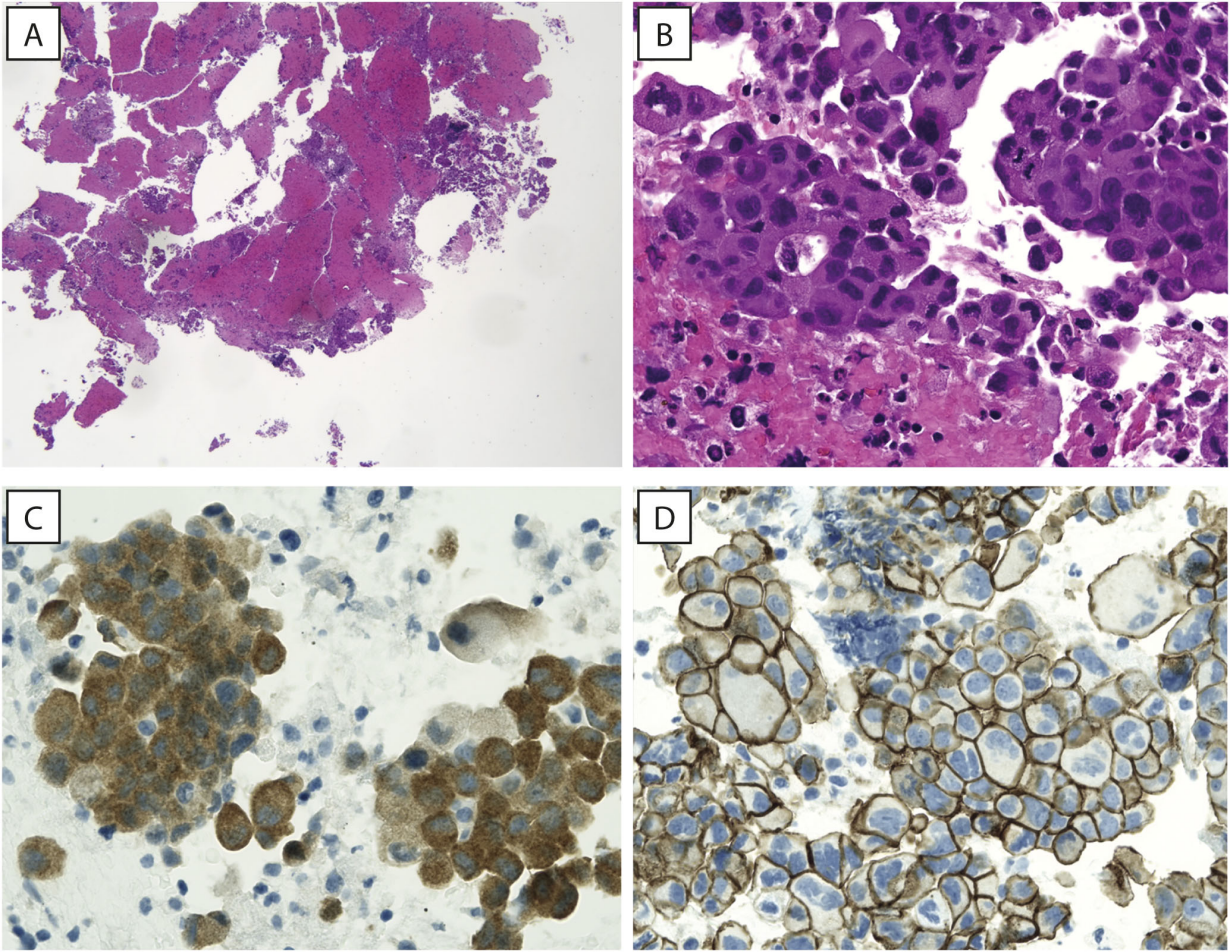
Hematoxylin and eosin images. **(A, B)** A low magnification view showing tissue fragments embedded in a clot (**A**, original magnification 40x) and a high magnification image showing papillary structures of adenocarcinoma, with neoplastic cells showing abundant eosinophilic cytoplasm and large, hyperchromatic nuclei (**B**, original magnification 400x). **(C)** Neoplastic cells showing strong cytoplasmic staining for BRAF (IHC for BRAF^V600E^, original magnification 400x). **(D)** Immunohistochemistry for beta-catenin (Beta-cat4), showing strong and diffuse membrane staining, while no positivity can be appreciated in the nuclei (original magnification 400x).

In January 2023, the patient underwent endobronchial ultrasound-guided transbronchial needle aspiration fibro bronchoscopy on the right paratracheal mediastinal lymph node and on a lung lesion of the anterior segment of the right lower lobe. Histological examination revealed an NSCLC histotype (**Figures 2A, B**). TNM tumor stage was IV.

Comprehensive genomic profiling using next-generation sequencing (NGS; Illumina TruSight™ Oncology 500 High-Throughput assay) allowed the detection of the V600E (missense variant c.1799T>A) mutation in the *BRAF* gene, supported by immunohistochemical evidence (**Figure 2C**) with strong clinical significance, a copy number loss in *CHEK2* gene, a missense variant of the *TP53* gene and a frameshift variant in *MAPK3* gene; several variants of unknown significance (VUS) in different genes, including a missense mutation in a microsatellite-stable (MSS) background of *RNF43* gene [NM_017763.4(RNF43):c.1112G>T (p.R371L)], were also reported. Immunohistochemistry for β-catenin showed strong and diffuse membrane staining, while no positivity was reported in the nuclei (**Figure 2D**). PD-L1 expression was assessed by immunohistochemistry using the DAKO assay and revealed a Tumor Proportion Score (TPS) of 95%. The patient was classified as MSS, and a medium TMB (6.3 mut/Mb) was detected.

In February 2023, the patient started the combination treatment with dabrafenib (75 mg tablets; two tablets at 10 a.m. and two tablets at 6 p.m.; 300 mg total daily dosage) and trametinib (2 mg, one tablet per day). From the beginning of the therapy, the patient reported a clinical benefit with gradual resolution of cough episodes and dyspnea, which allowed the suspension of oxygen therapy. A contrast-enhanced cranio-thorax-abdomen CT scan performed after 1 month of therapy reported a sharp reduction in lung cancer and hilo-mediastinal lymphadenomegaly; the central colliquation of the left adrenal metastasis was also reported.

The cranio-thorax-abdomen CT scan of July 2023 (after 5 months of therapy) reported a further sharp reduction in neoformation and hilo-mediastinal lymphadenomegaly and the disappearance of the left adrenal metastasis, while a right adrenal lesion was first identified (**Figure 1**, T2). In November 2023, after 9 months of therapy, the cranio-thorax-abdomen CT scan with contrast medium confirmed the reduction of the lung lesion, with residual scarring component; the right adrenal lesion was not visible, and the contralateral lesion was stable (**Figure 1**, T3).

**Figure 3:**
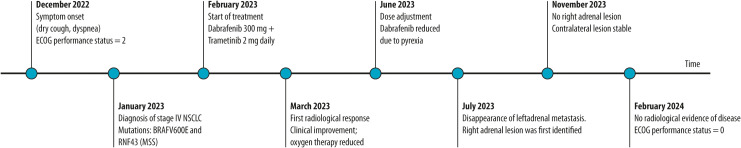
Clinical timeline of the patient. The timeline illustrates symptom onset, diagnosis, initiation of targeted therapy (dabrafenib plus trametinib), radiological and clinical responses, dose adjustment due to pyrexia, and subsequent disease control. Complete radiological remission and performance status improvement were observed by February 2024.

Episodes of fever and hyperpyrexia occurred immediately after starting therapy; treatment with paracetamol was necessary with the periodic and repeated suspension of the dabrafenib and trametinib combination treatment. For this reason, starting from June 2023, the dosage of dabrafenib was gradually reduced as per schedule, up to the daily dosage of 200 mg. With this dosage reduction, the episodes of hyperpyrexia gradually stopped, allowing the patient to continue the treatment. At the last follow-up (February 2024), the clinical condition of the patient was good; the ECOG PS was zero, she was autonomous, and oxygen therapy was not necessary. No radiological evidence of oncological disease was reported; both adrenal lesions (left and right) were no longer detectable on imaging, and the primary lung lesion showed only residual scarring (**Figure 1**, T4). The clinical timeline of the patient is summarized in **Figure 3**.

## Discussion

Several tools, such as NGS and RNA sequencing, have greatly improved the capacity to detect predictive and prognostic molecular alterations. Therefore, detecting gene mutations, amplifications, and fusions has altered the history of several oncological diseases in both localized and metastatic settings (1).

Our clinical case represents the first report of marked efficacy of the dabrafenib–trametinib combination, reported in an 85-year-old patient diagnosed with NSCLC with the presence of MSS, *BRAF* ^V600E^ and *RNF43* mutations. *RNF43* mutations include frameshift, nonsense, and missense variants, each with distinct functional implications. In MSI tumors, *RNF43* mutations are often frameshift alterations due to defective mismatch repair, leading to loss-of-function. In contrast, our patient had a missense mutation in a microsatellite-stable (MSS) background. This type of mutation may retain partial function or exert context-specific effects. Pyrexia during the combination treatment is the most reported adverse event in clinical trials and was the only adverse event observed in our patient (5). Previous experience with combination therapy in patients with *BRAF^V600E^*-mutant melanoma suggests that most grade 3 or 4 adverse events can be managed through dose modification; in our patient, this toxicity was successfully managed through a dose reduction. This case report also attests to the feasibility of the treatment in terms of quality of life, even in older patients.

The analysis of the expression pattern and mutation signature of *RNF43* suggests that this gene can be reported as an important prognostic biomarker in pan-cancer (11). Furthermore, *RNF43* appears to be a critical modulator in the tumor immune microenvironment, resulting in a promising biomarker for predicting the efficacy of immunotherapeutic regimens (11). Literature data suggest the hypothesis of a possible cross-talk between MAPK and RNF43 pathways in modulating the anti-tumor activity of *BRAF* ^V600E^-targeted treatments, also related to the state of microsatellites (10). In addition, the results of a recent *in vitro* study reported that *RNF43*-mutated pancreatic cancer cells show elevated B-RAF/MEK activity and are highly sensitive to MEK inhibitors (12). Additionally, in colorectal cancer, *RNF43* mutations have been shown to predict response to combined anti-BRAF/EGFR therapies (10), further highlighting the therapeutic relevance of this alteration across tumor types. For instance, in colorectal cancer, *BRAF^V600E^* mutations often co-occur with *RNF43* mutations and infrequently with *APC* mutations. These characteristics correlate with specific clinical and molecular profiles, such as right-sided tumor localization (10, 13). This highlights the potential relevance of *RNF43* as a biomarker in other tumor contexts. Therefore, future research should explore incorporating this biomarker into routine testing, along with *BRAF* and MSI status, and evaluate their integration with other transcriptomic, microbiome, or microenvironmental indicators to optimize the clinical management of patients with *BRAF* ^V600E^ NSCLC. Of note, we found that β-catenin was expressed only on the cell membrane and not in the cytoplasm and/or nucleus, in agreement with evidence reported by Elez et al. (10); this suggests that also in our patient the response to therapy was independent of β-catenin signaling and involved non-canonical WNT pathways activated by *RNF-43* mutations (14–16).

We acknowledge the limitations of our findings due to their derivation from a case report and the consequent need to supplement them with other types of evidence for a more comprehensive understanding of proposed mechanisms. At the same time, our case report proposes for the first time the association between enhanced efficacy of the dabrafenib-trametinib combination and *RNF43* mutations in lung cancer, further suggesting the hypothesis on the relevance of *RNF43* mutations in predicting the clinical benefit of targeted therapies and on the cross-talk between the MAPK and WNT pathways that may modulate the anti-tumor activity of anti-BRAF therapies (10).

## Conclusion

This case report suggests that *RNF43* mutations represent a promising biomarker that warrants further validation for its potential in helping prioritize therapy combinations in selected lung cancer patients with *BRAF* ^V600E^ who are most likely to benefit and in identifying those patients for whom alternative treatment approaches are needed.

## Data availability statement

The raw data supporting the conclusions of this article will be made available by the authors, without undue reservation.

## Ethics statement

The study was conducted within the protocol approved by the Ethics Committee of “Agostino Gemelli” University Hospital Foundation (Prot. N. 0001151/24 –28/06/2024). Written informed consent was obtained from the participant/patient(s) for the publication of this case report.
